# Prognostic value of FOXM1 in solid tumors: a systematic review and meta-analysis

**DOI:** 10.18632/oncotarget.15764

**Published:** 2017-02-27

**Authors:** Lijun Li, Dang Wu, Qun Yu, Lingdi Li, Pin Wu

**Affiliations:** ^1^ Department of Surgery, Hangzhou Xixi Hospital, Hangzhou, China; ^2^ Department of Radiation Oncology, Second Affiliated Hospital, Zhejiang University School of Medicine, Zhejiang University, Hangzhou, China; ^3^ Fourth Ward of Neurosurgery, Division of Nursing, Second Affiliated Hospital, Zhejiang University School of Medicine, Zhejiang University, Hangzhou, China; ^4^ Department of Oncology, Hangzhou Cancer Hospital, Hangzhou, China; ^5^ Department of Thoracic Surgery, Second Affiliated Hospital, Zhejiang University School of Medicine, Zhejiang University, Hangzhou, China

**Keywords:** FOXM1, solid tumors, prognosis, overall survival, disease free survival

## Abstract

Accumulated studies have provided controversial evidences of the association between Forkhead Box M1 (FOXM1) expression and survival of human solid tumors. To address this inconsistency, we performed a meta-analysis with 23 studies identified from PubMed and Medline. We found elevated FOXM1-protein expression was significantly associated with worse 3-year overall survival (OS) (OR = 3.30, 95% CI = 2.56 to 4.25, *P* < 0.00001) 5-year OS (OR =3.35, 95% CI = 2.64 to 4.26, *P* < 0.00001) and 10-year OS (OR = 5.24, 95% CI = 2.61 to 10.52, *P* < 0.00001) of human solid tumors. Similar results were observed when disease free survival (DFS) were analyzed. Subgroup analysis showed that FOXM1 overexpression was associated with poor prognosis of colorectal cancer, gastric cancer, hepatic cancer, lung cancer and ovarian cancer. High expression level of FOXM1 was also associated with advanced tumor stage. In conclusion, elevated FOXM1 expression is associated with poor survival in most solid tumors. FOXM1 is a potential biomarker for prognosis prediction and a promising therapeutic target in human solid tumors.

## INTRODUCTION

Forkhead Box M1 (FOXM1), a transcription factor of the forkhead family, is well demonstrated to be critical for proliferation, apoptosis, migration and invasion of human cancer [1]. FOXM1 is also linked to angiogenesis, cellular senescence, DNA damage response, drug resistance, cancer stem cell renewal and differentiation of cancer [2]. Recent study demonstrate the genic overexpression of FOXM1 is a major predictor of adverse outcomes across 39 human malignancies by a computational analysis of FOXM1 expression in mRNA level [3]. Some studies have demonstrated FOXM1-targeted therapy could effectively restrain tumor development of cancer [4–7]. These evidences suggest FOXM1 may be an attractive prognostic prediction biomarker and therapeutic target for human cancers. However, the prognostic value of FOXM1-protein in human solid tumors is still controversial.

A plenty of studies showed that elevated FOXM1 expression in tumor tissue was correlated with poor survival of patients with various solid tumors such as angiosarcoma [8], breast cancer [9], cervical cancer [10], colorectal cancer [11–13], gastric cancer [14–16], hepatic cancer [17, 18], laryngeal squamous cell cancer [19], lung cancer [20–22], malignant peripheral nerve sheath tumor [23], medulloblastoma [24], esophageal cancer [25], ovarian cancer [26, 27], pancreatic cancer [28, 29] and renal cell cancer [30]. However, other study showed there is no significantly correlation between FOXM1 expression in tumor tissue and outcome of patients with esophageal cancer [31].

Therefore, we carried out a meta-analysis combining available evidences to evaluate the prognostic value of FOXM1 expression in solid tumors. We also evaluated whether the correlation between FOXM1 expression and outcome of patients is different between tumor types. This meta-analysis intended to assess the role of FOXM1 in relation to survival in solid tumors, thereby shed more light on the development of FOXM1-targeted therapy and prognostic prediction in solid tumor.

## RESULTS

### Search results and study characteristics

**Figure 1:**
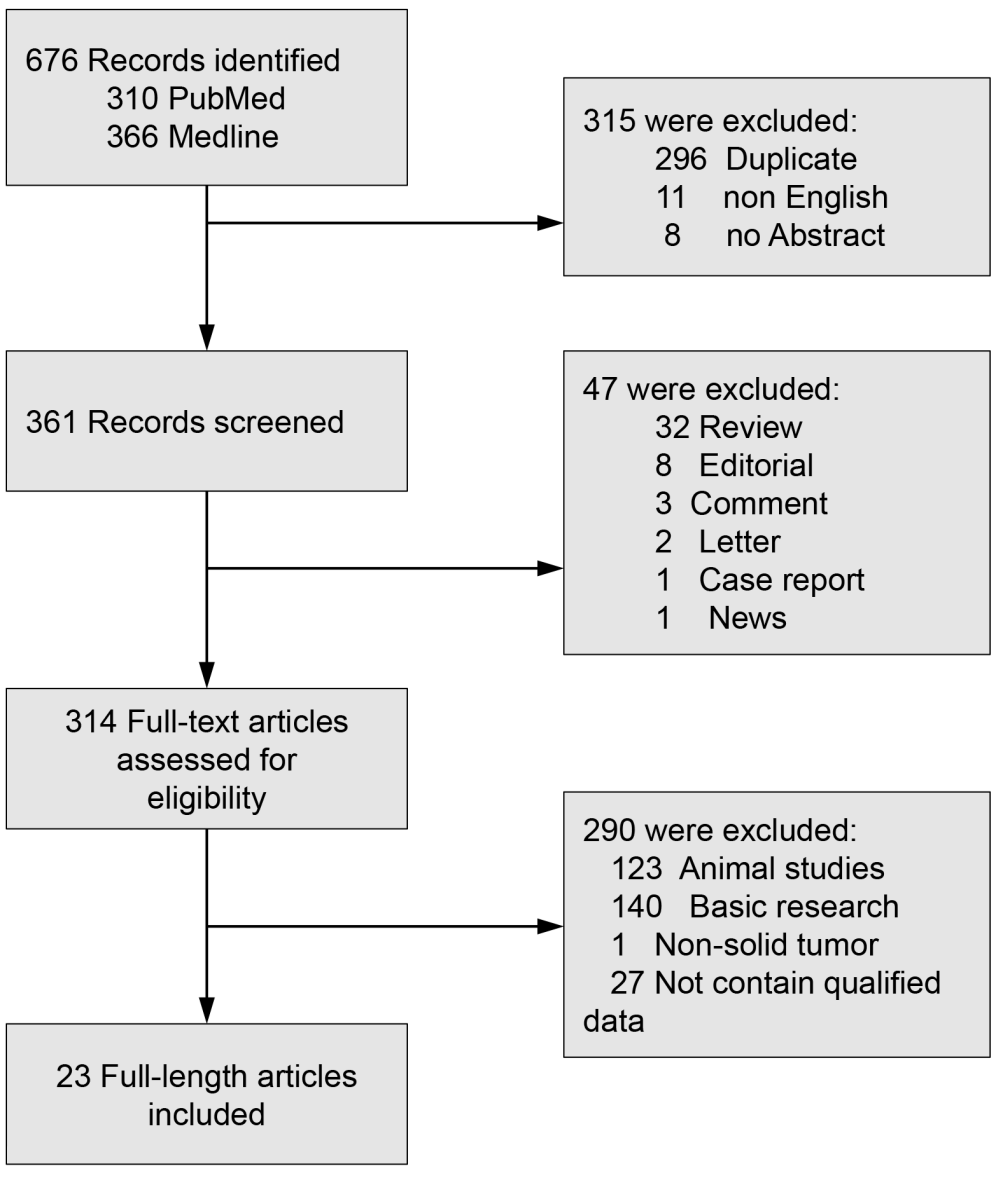
Flow diagram of study selection FOXM1: Forkhead Box M1.

Twenty-three studies with a total of 2847 patients were included (Figure 1). Three evaluated gastric cancer [14–16], three evaluated lung cancer [20–22], two studies evaluated colorectal cancer [11, 12], two evaluated hepatocellular cancer [17, 18], two evaluated esophageal cancer [25, 31], two evaluated ovarian cancer [26, 27], two evaluated pancreatic cancer [28, 29], one evaluated breast cancer [9], angiosarcoma [8], cervical cancer [10], laryngeal squamous cell cancer [19], malignant peripheral nerve sheath tumor [23], medulloblastoma [24] and renal cell cancer [30] respectively. For the region, 20 studies were conducted in Asia, 2 studies in Europe, 1 studies in America.

### Evaluation and expression of FOXM1

The major information of included studies and extracted data from the included studies were summarized in Table 1. All studies detected FOXM1 expression by IHC. There are seventeen studies used antibody of FOXM1 with clone sc-502, one study used clone K-19 and four studies did not report the antibody clone. The mean of FOXM1 positive patients in solid tumors was 56.71%, range from 25% to 78.64%.

**Table 1:** Characteristics of studies included in the meta-analysis

Ref	PMID	Type of tumor	Patient No.	Age, median (range)	Male/ Female	Stage	FOXM1 (+/−) NO.	3-year OS (+/−)%	5-year OS (+/−)%	10-year OS (+/−)%	NOS Score
Bektas, N., et al. (2008)	18254960	Breast cancer	204	56(25-82)	NR	I-III	146/56	80.84/90.92	68.57/80	53.46/67.3	8
Chu, X.-Y., et al. (2012)	22326401	Colorectal cancer	112	NR	36/76	I-IV	57/55	44.64/63.73	44.64/55.36	NR	7
He, S.-y., et al. (2012)	22943878	Cervical cancer	102	43.55(26-72)	0/102	I-II	75/27	63.33/82.22	54.07/82.22	NR	8
Hu, C.-J., et al. (2015)	25482013	Gastric cancer	40	NR	28/12	I-IV	26/14	44.32/62.37	NR	NR	6
Hui, M. K. C., et al. (2012)-C	21976009	Oesophageal cancer	64	NR	47/17	I-IV	27/37	18.5/18.5	9/18.5	NR	6
Hui, M. K. C., et al. (2012)-N	21976009	Oesophageal cancer	64	NR	47/17	I-IV	16/48	15.98/19.07	15.98/12.89	NR	6
Ito, T., et al. (2016)	27162541	Angiosarcoma	107	66.3	63/44	I-IV	28/66	35.34/65.97	23.82/47.12	0/37.7	8
Jiang, L.-Z., et al. (2011)	21334713	Laryngeal squamous cell carcinoma	89	59(33-85)	78/11	I-IV	49/40	76.33/89.61	57.97/77.78	NR	8
Kong, F.-F., et al. (2014)	24715097	Lung cancer	68	59.44	38/30	I-IV	43/25	36.53/72.02	11.9/63.05	NR	7
Li, D., et al. (2013)	23136192	Colorectal cancer	203	NR	86/117	I-IV	132/71	80.31/92.91	49.6/85.9	NR	6
Li, X., et al. (2014)	23873251	Gastric cancer	103	NR	68/35	I-IV	81/22	55.34/77.12	35.73/63.4	NR	8
Ning, Z., et al. (2014)	24993031	Pancreatic ductal adenocarcinoma	136	67(34-80)	74/62	II	86/50	29.33/39.00	10.56/26.1	2.64/23.46	8
Okada, K., et al. (2013)	23054116	Gastric cancer	77	NR	52/25	NR	53/24	70.2/90.26	59.6/90.26	NR	7
Priller, M., et al. (2011)	21918172	Medulloblastoma	41	NR	22/19	NR	20/21	42.98/90.73	42.98/85.11	22.75/84.83	6
Sun, H.-C., et al. (2011)	21431285	Hepatocellular cancer	151	NR	130/20	I-III	89/61	32.76/76.85	32.76/64.29	NR	8
Takata, A., et al. (2014)	24778055	Oesophageal cancer	174	64(46-81)	155/19	I-IV	94/80	44.16/67.51	42.39/64.47	36.8/61.68	8
Wen, N., et al. (2014)	24885308	Ovarian cancer	158	53(26-79)	0/158	I-IV	101/57	44.37/68.49	13.83/41.16	NR	8
Wu, X.-R., et al. (2013)	23263830	Renal cell cancer	87	56.6(24-82)	60/27	NR	37/50	69.66/95.21	69.66/79.04	NR	8
Xia, J.-T., et al. (2012)	22249132	Pancreatic ductal adenocarcinoma	80	59(28-78)	48/32	I-IV	53/27	0/51.65	NR	NR	8
Xia, L., et al. (2012)	22613004	Hepatocellular cancer	406	NR	331/75	I-III	201/105	41.89/75.52	27.14/54.28	NR	8
Xu, N., et al. (2013)	23536876	Lung cancer	175	NR	122/53	I-IV	112/63	37.58/84.19	27.1/73.1	NR	8
Yang, D. K., et al. (2009)	19121844	Lung cancer	69	62(43-84)	65/4	I-III	26/43	51.3/76.84	32.14/76.65	NR	9
Yu, J., et al. (2011)	21325289	Malignant peripheral nerve sheath tumor	82	NR	NR	NR	34/48	20.51/56.2	13.68/40.81	7.28/28.91	6
Zhao, F., et al. (2014)	25411964	Ovarian cancer	119	NR	NR	I-IV	53/29	76.36/81.56	58.44/77.4	35.58/77.4	6

FOXM1: Forkhead Box M1; 1: Cohort 1; 2: Cohort 2; 3: Cohort 3; C: cytoplasmic expression; N: nuclear expression; OS: Overall survival.

### Association of FOXM1 with OS

**Figure 2:**
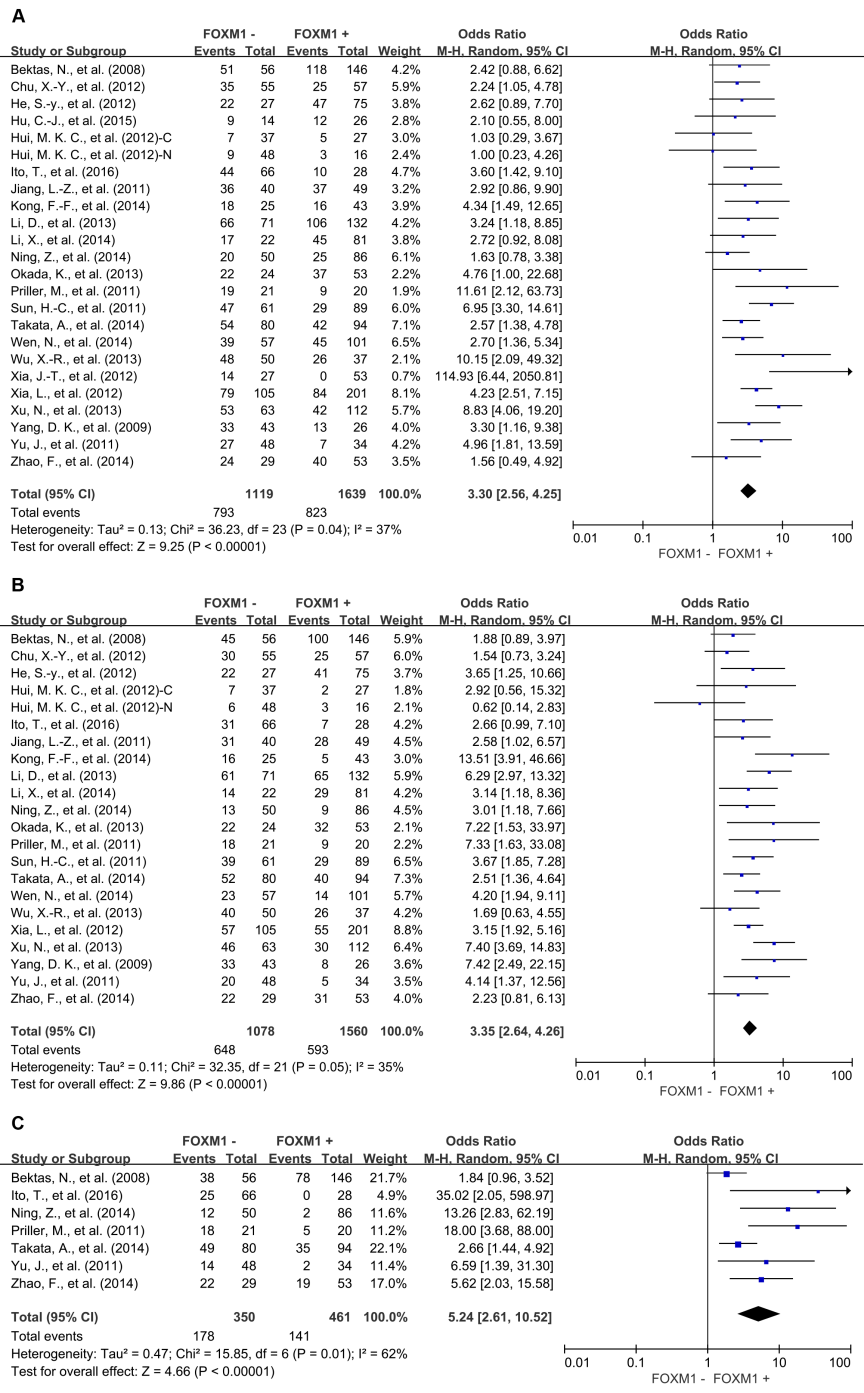
Overall survival (OS) according to FOXM1 expression in tumor tissue **A**. 3-year OS; **B**. 5-year OS; **B**. 10-year OS. C: cytoplasmic expression; N: nuclear expression.

**Figure 3:**
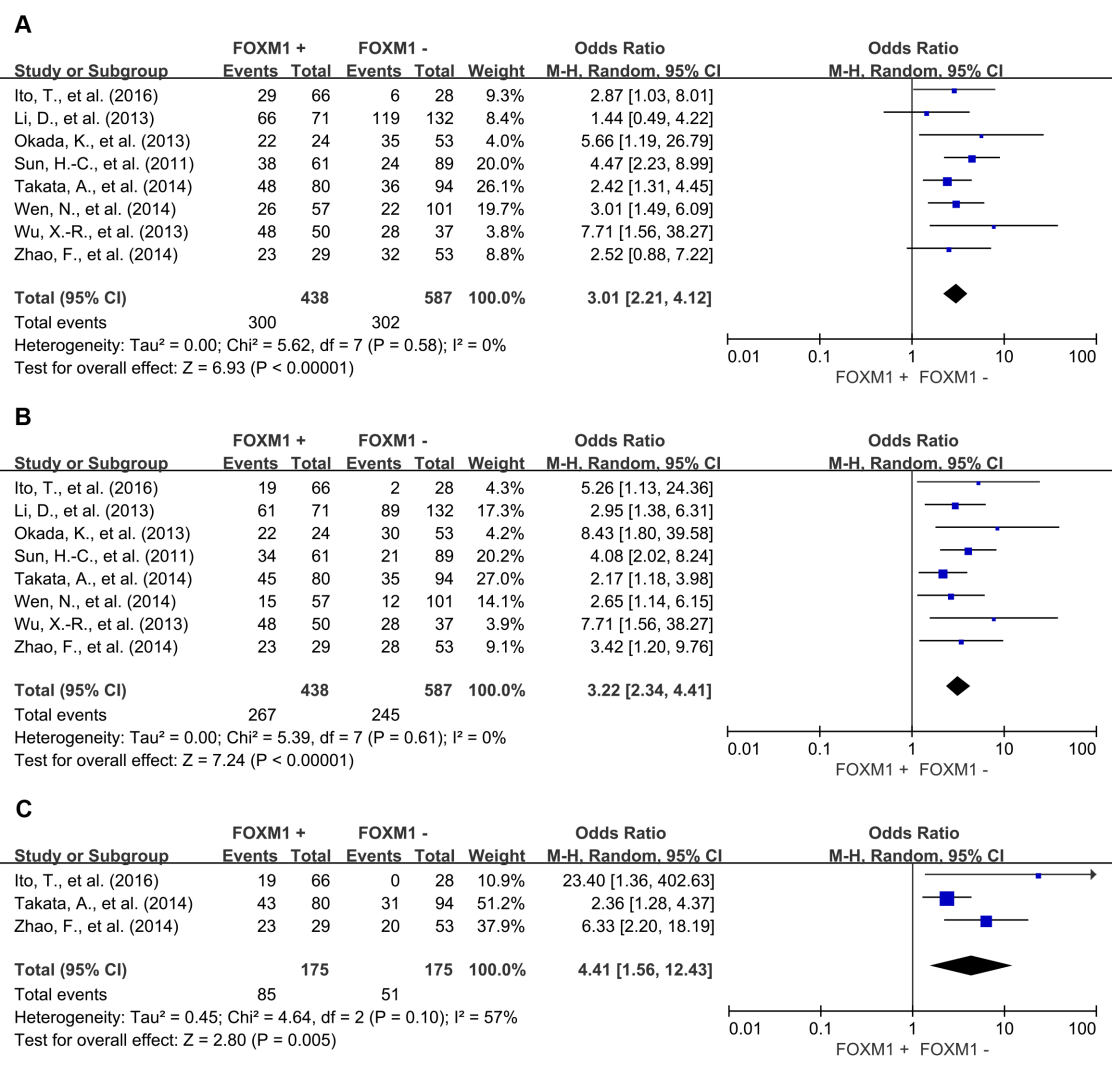
Disease free survival (DFS) according to FOXM1 expression in tumor tissue **A**. 3-year DFS; **B**. 5-year DFS; **B**. 10-year DFS.

The combined analysis of 23 studies showed that FOXM1 overexpression in tumor tissue was associated with worse 3-year OS (OR = 3.30, 95% CI = 2.56 to 4.25, *P* < 0.00001), 5-year OS (OR = 3.35, 95% CI = 2.64 to 4.26, *P* < 0.00001) and 10-year OS (OR = 5.24, 95% CI = 2.61 to 10.52, *P* < 0.00001) of solid tumors (Figure 2). Results of 8 studies showed that FOXM1 expression was associated with statistically significant poor 3-year DFS (OR = 3.01, 95% CI = 2.21 to 4.12, *P* < 0.00001), 5-year DFS (OR = 3.22, 95% CI = 2.34 to 4.41, *P* < 0.00001) and 10-year DFS (OR = 4.41, 95% CI = 1.56 to 12.43, *P* = 0.005) (Figure 3).

**Figure 4:**
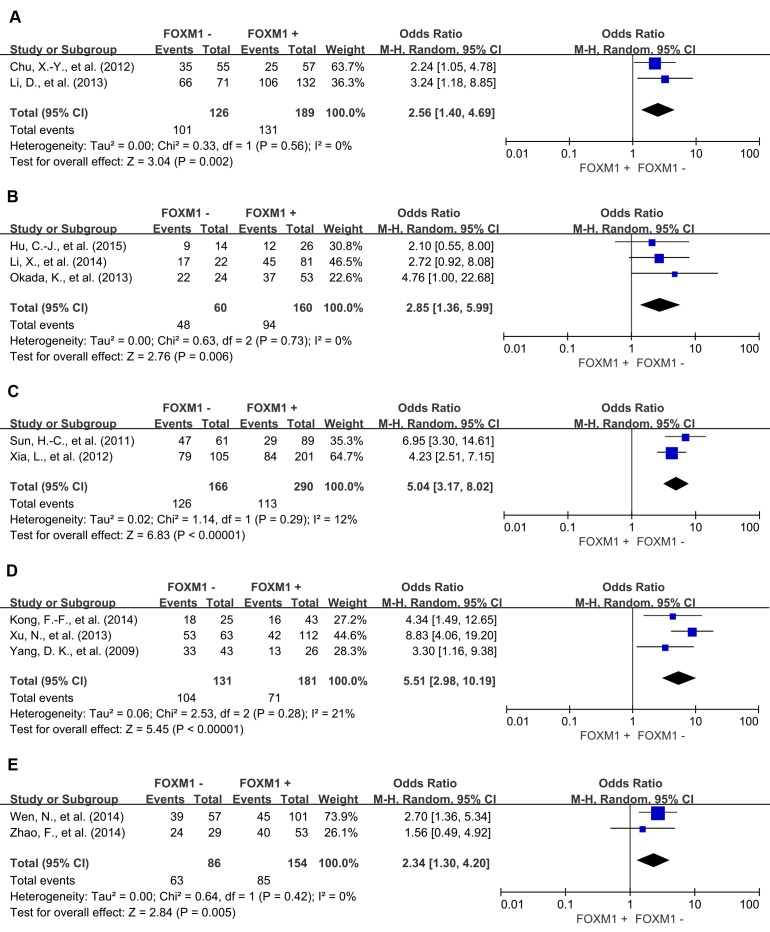
Subgroup analysis of 3-year OS according to FOXM1 expression in different tumor types **A**. colorectal cancer; **B**. gastric cancer; **C**. hepatic cancer; **D**. lung cancer; **E**. ovarian cancer.

**Figure 5:**
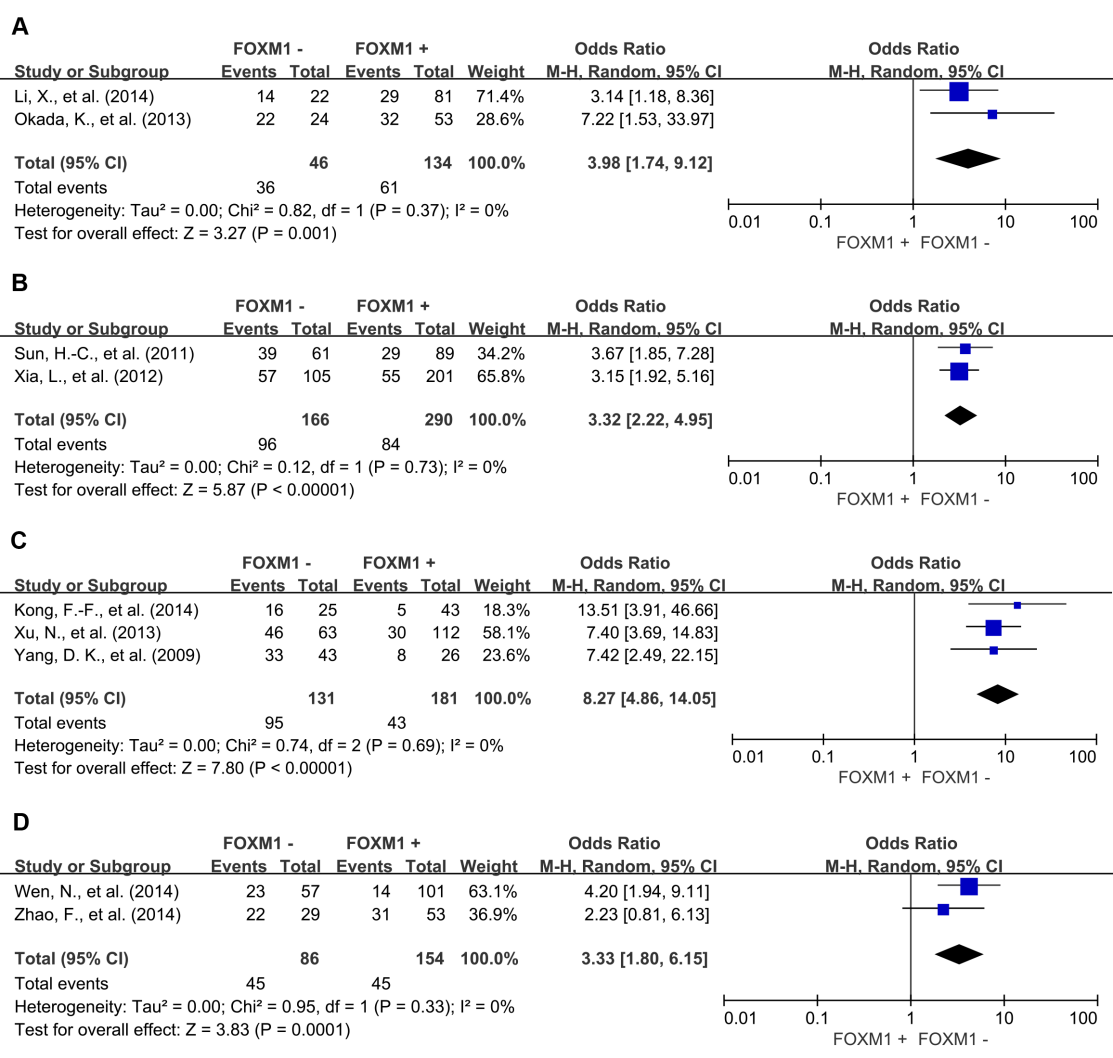
Subgroup analysis of 5-year OS according to FOXM1 expression in different tumor types **A**. gastric cancer; **B**. hepatic cancer; **C**. lung cancer; **D**. ovarian cancer.

In the stratified analysis by tumor types, FOXM1 expression was associated with worse 3-year OS of colorectal cancer (OR = 2.56, 95% CI = 1.40 to 4.69, *P* = 0.002), gastric cancer (OR = 2.85, 95% CI = 1.36 to 5.99, *P* = 0.006), hepatic cancer (OR = 5.04, 95% CI = 3.17 to 8.02, *P* < 0.00001), lung cancer (OR = 5.51, 95% CI = 2.98 to 10.19, *P* < 0.00001) and ovarian cancer (OR = 2.34, 95% CI = 1.30 to 4.20, *P* = 0.005) (Figure 4). Consistent with this 3-year OS, FOXM1 expression was associated with worse 5-year OS of gastric cancer (OR = 3.98, 95% CI = 1.74 to 9.12, *P* = 0.001), hepatic cancer (OR = 3.32, 95% CI = 2.22 to 4.95, *P* < 0.00001), lung cancer (OR = 8.27, 95% CI = 4.86 to 14.05, *P* < 0.00001) and ovarian cancer (OR = 3.33, 95% CI = 1.80 to 6.15, *P* = 0.0001) (Figure 5). There was no significant association between FOXM1 expression and survival of esophageal cancer and pancreatic cancer (Supplementary Figure 1).

**Figure 6:**
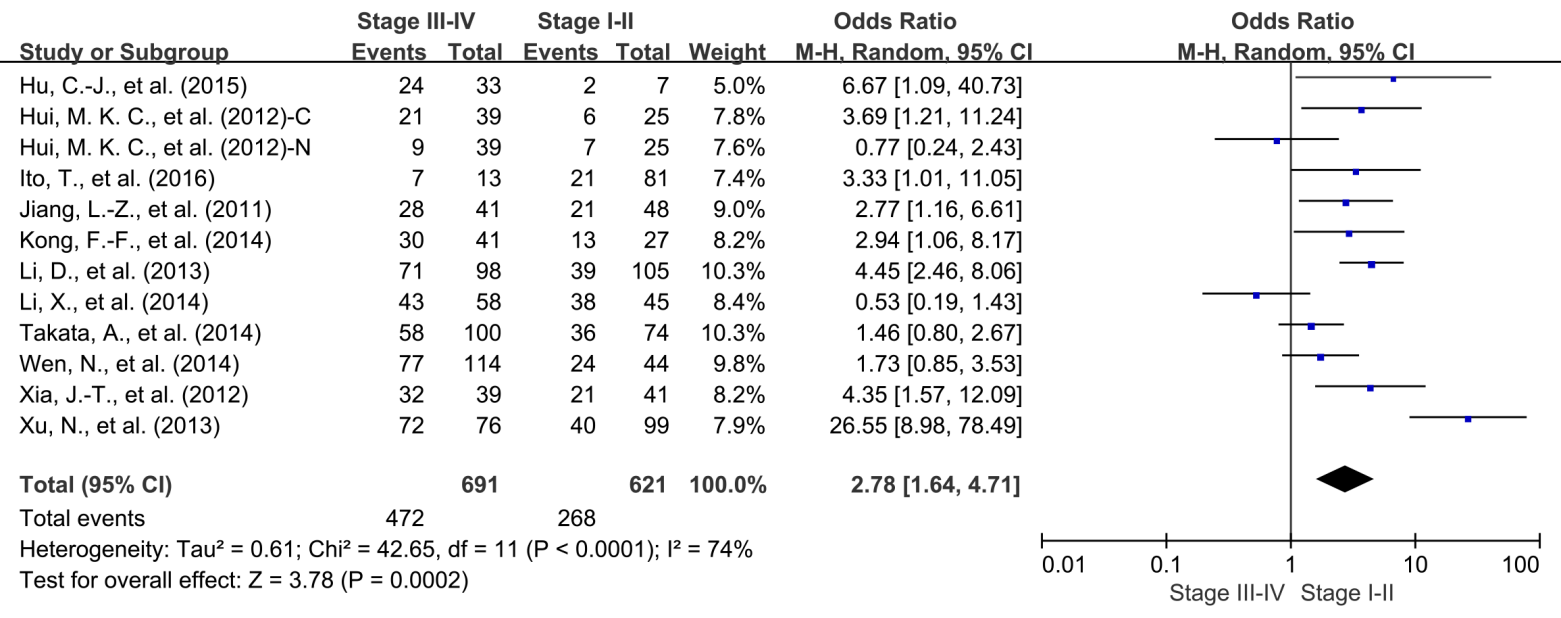
Correlation of FOXM1 expression and tumor stage C: cytoplasmic expression; N: nuclear expression.

We also evaluated the correlation between FOXM1 expression and the TNM stage of tumor. High expression level of FOXM1 was significantly associated with advanced TNM stage (OR = 2.78, 95% CI = 1.64 to 4.71, *P* = 0.0002) (Figure 6). Moreover, we found that FOXM1 overexpression in tumor tissue was also significantly correlated with the 3-year OS (OR = 2.62, 95% CI = 1.62 to 4.26, *P* < 0.0001) and 5-year OS (OR = 3.12, 95% CI = 1.56 to 6.28, *P* = 0.001) of patients with cancer in early stage (Supplementary Figure 2).

Meta-regression analysis showed that publication year, country, gender and NOS score did not contribute to the heterogeneity (data not shown).

### Sensitivity analyses

Removal of the studies that was an outlier (score, IRS, > 50%) or no report (NR) with regard to the cutoff of FOXM1 overexpression did not influence results for 3- or 5-year OS (OR = 3.79, 95% CI = 2.89 to 4.97, *P* < 0.00001; OR = 3.19, 95% CI = 2.54 to 4.01, *P* < 0.00001; respectively). Exclusion of these studies did not reduce heterogeneity for 3- or 5-year OS (Cochran's Q *P* = 0.10, I^2^ = 31%; Cochran's Q *P* = 0.24, I^2^ = 19%, respectively).

Removal of studies with NOS score 6 did not influence results for 3- or 5-year OS (OR = 3.57, 95% CI = 2.70 to 4.72, *P* < 0.00001; OR = 3.32, 95% CI = 2.56 to 4.30, *P* < 0.00001, respectively). Exclusion of these studies did not reduce heterogeneity for 3- or 5-year OS (Cochran's Q *P* = 0.07, I^2^ = 37%; Cochran's Q *P* = 0.08, I^2^ = 35%, respectively).

### Publication bias

Funnel plot analysis showed that there was no statistical evidence of publication bias in our meta-analysis (data not shown).

## DISCUSSION

This meta-analysis is the most comprehensive assessment of the literatures regarding FOXM1-protein expression and tumor prognosis to date. We systematically evaluated survival data for 2847 solid tumor patients included in 23 different studies. Our study demonstrated that the expression of FOXM1 is a potential biomarker of poor prognosis in most solid tumors, with consistent results of both OS and DFS at 3, 5 and 10 years. Regarding to the tumor types, elevated FOXM1 expression in tumor tissues were associated with worse OS of most human solid tumors, such as colorectal cancer, gastric cancer, hepatic cancer, lung cancer and ovarian cancer. But FOXM1 expression is not statistical correlation with survival of esophageal cancer and pancreatic cancer. In addition, we found expression level of FOXM1 was significantly associated with advanced stage of human solid tumors. And FOXM1 overexpression in tumor tissue is also correlated with unfavorable outcome in early stage cancers.

FOXM1 is a key transcription factor, which provides a balanced transcriptional programme to ensure proper growth and maturation during embryogenesis and foetal development as well as to manage appropriate homeostasis and repair of adult tissues [32]. However, deregulated FOXM1 signalling in cancer is involved in cell migration [33, 34], invasion [35, 36], angiogenesis [37, 38], stem cell renewal [39, 40], DNA damage repair and cellular senescence [41, 42], which impact tumor initiation [43, 44], progression, metastasis, angiogenesis and drug resistance [45]. These evidences suggest that dysregulated of FOXM1 expression and FOXM1 signal pathway in tumor microenvironment may serve as a key factor in human cancer development.

Recently, a comprehensive genomics-based study included 18,000 patients demonstrated that elevated FOXM1 gene expression is correlated with adverse outcome across 39 malignancies [3]. Consistently, our meta-analysis found overexpression of FOXM1 in protein level is also significantly correlated with poor outcome of human solid tumors. These evidences highlight the potential of FOXM1 to be a valuable therapeutic target and prognostic biomarker for solid tumor.

In summary, FOXM1 expression in solid tumor tissues is associated with poor survival in most solid tumors, which suggests that FOXM1 is a valuable prognostic biomarker and a promising therapeutic target for solid tumors.

## MATERIALS AND METHODS

This meta-analysis was conducted according to the statement for reporting systematic reviews and meta-analyses [46]. This study summarized and analyzed the results of previous studies, so the ethical approval was not necessary.

### Search strategy and study selection

An electronic search of Pubmed and Web of Science were undertaken for studies evaluating FOXM1 expression and clinical outcome in solid tumors from 1994 to June 2016. The search was performed with subject heading terms including “FOXM1 protein, human” and “Neoplasms” and the results were limited to human studies of solid tumors. A total of 310 and 366 entries were identified, respectively. Inclusion criteria were the measurement of FOXM1 expression in tumor tissues by immunohistochemistry (IHC), availability of survival data for at least 3 years, and original article written in English. Citation lists of retrieved articles were manually screened to ensure sensitivity of the search strategy. Study selection was based on the association of FOXM1 expression and survival. Two reviewers (Lijun Li and Dang Wu) evaluated independently all of the full articles for study eligibility. Inter-reviewer agreement was assessed using Cohen's kappa coefficient. Disagreement was resolved by consensus.

### Data extraction

Overall survival (OS) and disease free survival (DFS) were the primary endpoints of interest. Data were extracted using predefined abstraction forms. The following details were extracted by two authors (Lijun Li and Dang Wu): name of first author, year of publication, country of publication, tumor type, patient number, tumor stage, antibodies used for the evaluation, method and score for FOXM1 assessment, and cut-off values to determine FOXM1 positivity. Data for 3, 5 and 10 year of OS and DFS were extracted from tables or Kaplan-Meier curves for both FOXM1 negative and FOXM1 positive group.

The studies included in our meta-analysis were all cohort studies. Two independent authors evaluated the quality of each included study using Newcastle-Ottawa Scale (NOS) [47]. The studies with 6 scores or more were considered as high quality studies. A consensus NOS score for each item was achieved finally.

### Data synthesis

The relative frequency of OS and DFS at 3, 5 and 10 years between FOXM1 negative and FOXM1 positive group was presented as an odds ratio (OR) and its 95% confidence interval (CI). Sensitivity analyses were carried out for different analytical methods and cut-offs for defining FOXM1 expression and NOS scores for quality assessment of included studies. Publication bias was assessed by visual inspection of the funnel plot.

### Statistical analysis

Data were extracted from the primary publications and combined into a meta-analysis using RevMan 5.3 analysis software (Cochrane Collaboration, Copenhagen, Denmark). Estimates of ORs were weighted and pooled using the Mantel-Haenszel random effect model. Statistical heterogeneity was assessed using the Cochran's Q and I^2^ statistics. Differences between subgroups were assessed using methods as previous described by Deeks et al [48]. Meta-regression analysis was conducted using Stata 12.0 software (StataCorp LP, College Station, TX). All statistical tests were two-sided, and statistical significance was defined as *P* < 0.05. No correction was made for multiple statistical testing.

## SUPPLEMENTARY MATERIALS FIGURES


