# Supplementary material for: Prognostic value of FOXM1 in solid tumors: a systematic review and meta-analysis
Source: Oncotarget. 2017 Feb 27;8(19):32298–308. doi: 10.18632/oncotarget.15764 (PMC5458285; doi:10.18632/oncotarget.15764)
Supplement: Supplementary file 1 [file oncotarget-08-32298-s001.pdf]

Prognostic value of FOXM1 in solid tumors: a systematic review and meta-analysis

Supplementary Material

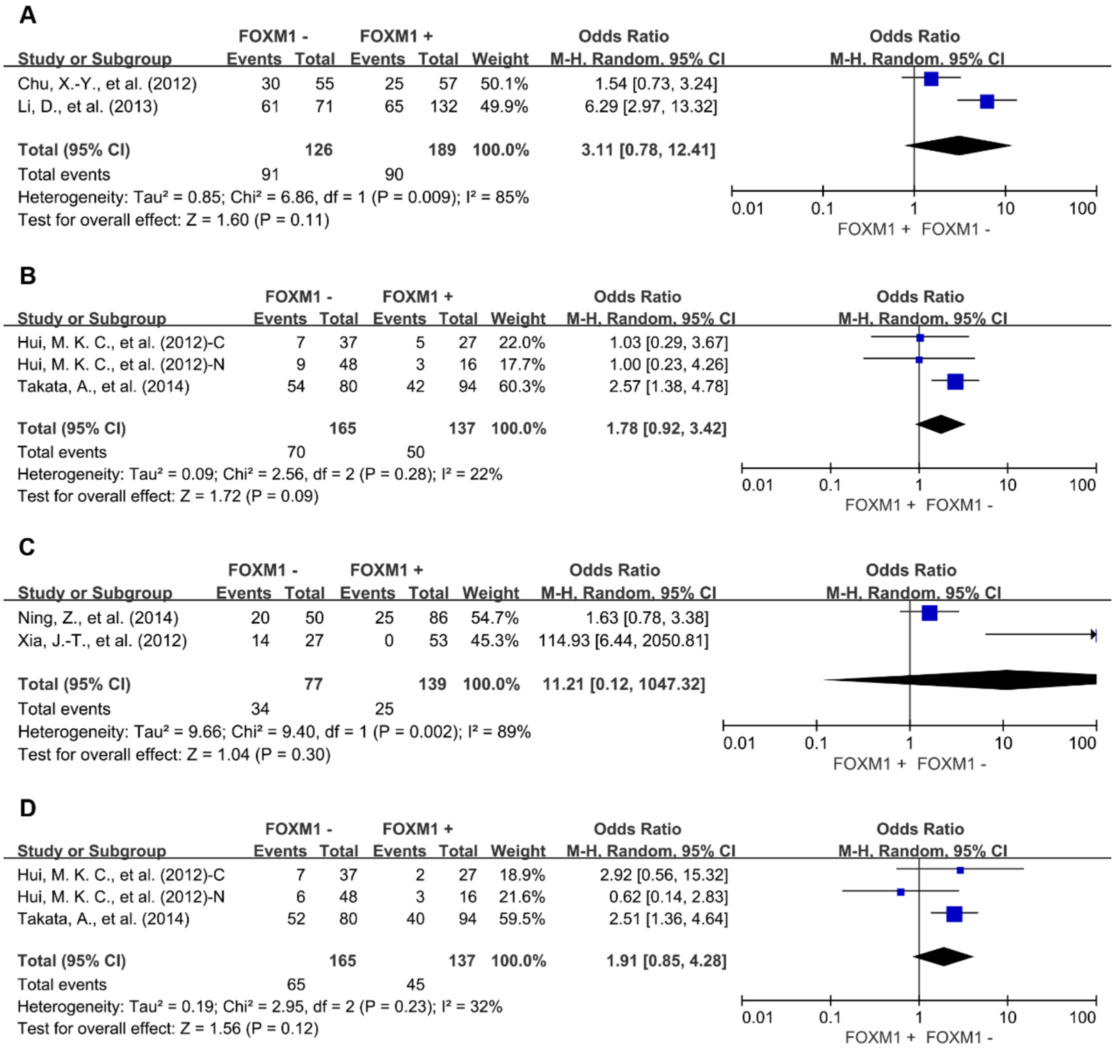

Supplementary Figure S1: Subgroup analysis of OS according to FOXM1 expression in different tumor types. (A) 3-year OS of colorectal cancer; (B) 3-year OS of esophageal cancer; (C) 3-year OS of pancreatic cancer; (D) 5-year OS of esophageal cancer. N: nuclear expression; C: cytoplasmic expression.

A

Study or Subgroup	FOXM1 + Events	FOXM1 + Total	FOXM1 - Events	FOXM1 - Total	Weight	Odds Ratio M-H, Random, 95% CI
He, S.-y., et al. (2012)	22	27	47	75	27.8%	2.62 [0.89, 7.70]
Ning, Z., et al. (2014)	20	50	25	86	34.8%	1.63 [0.78, 3.38]
Xia, J.-T., et al. (2012)	14	20	0	21	8.1%	95.92 [5.01, 1837.37]
Xu, N., et al. (2013)	51	59	27	40	29.4%	3.07 [1.13, 8.32]
**Total (95% CI)**		**156**		**222**	**100.0%**	**3.11 [1.23, 7.85]**
Total events	107		99			

Heterogeneity: Tau² = 0.50; Chi² = 7.79, df = 3 (P = 0.05); I² = 61%
Test for overall effect: Z = 2.40 (P = 0.02)

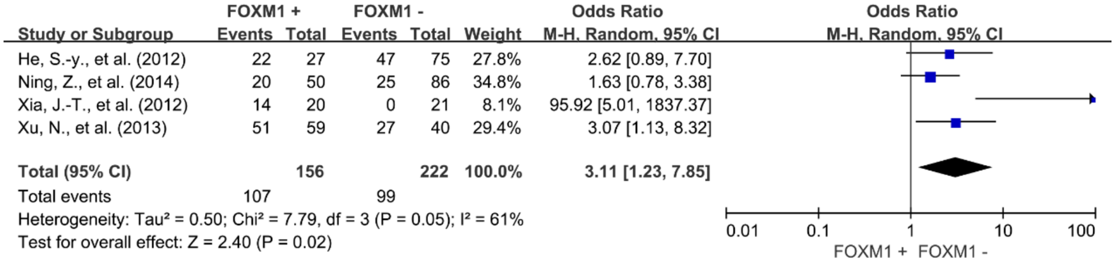

B

Study or Subgroup	FOXM1 + Events	FOXM1 + Total	FOXM1 - Events	FOXM1 - Total	Weight	Odds Ratio M-H, Random, 95% CI
He, S.-y., et al. (2012)	22	27	41	75	27.4%	3.65 [1.25, 10.66]
Ning, Z., et al. (2014)	13	50	9	86	32.4%	3.01 [1.18, 7.66]
Xia, J.-T., et al. (2012)	10	20	0	21	5.3%	43.00 [2.29, 806.44]
Xu, N., et al. (2013)	45	59	25	40	34.9%	1.93 [0.80, 4.64]
**Total (95% CI)**		**156**		**222**	**100.0%**	**3.12 [1.56, 6.28]**
Total events	90		75			

Heterogeneity: Tau² = 0.16; Chi² = 4.46, df = 3 (P = 0.22); I² = 33%
Test for overall effect: Z = 3.20 (P = 0.001)

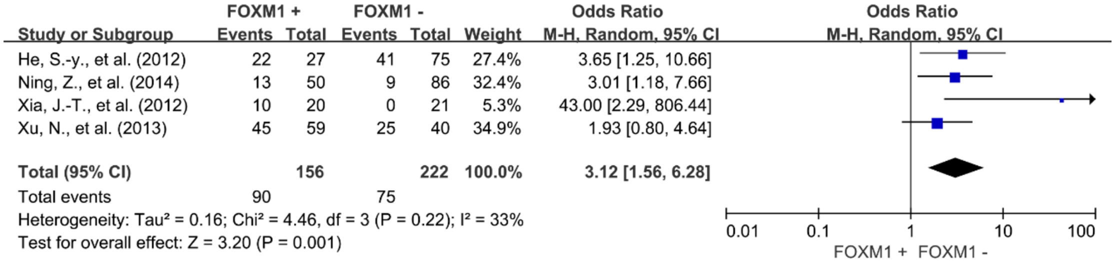

Supplementary Figure S2: OS according to FOXM1 expression in early stage cancers. (A) 3-year OS; (B) 5-year OS.
